# Preferences for Internet Plus Nursing Services Among Nurses and Patients in China: A Systematic Review of Discrete Choice Experiments

**DOI:** 10.1155/jonm/8852180

**Published:** 2026-10-04

**Authors:** Yue Li, Xuemei Xie, Hang Li, Yingxin Li, Xi Huang, Wenqian Su, Xiujuan Zhang, Yaohua Wu

**Affiliations:** ^1^ Department of Neonatology West China Second University Hospital Sichuan University Chengdu China scu.edu.cn; ^2^ Key Laboratory of Birth Defects and Related Diseases of Women and Children Ministry of Education Chengdu Sichuan China meb.gov.tr; ^3^ Sichuan Provincial Center for Mental Health Sichuan Provincial People’s Hospital School of Medicine University of Electronic Science and Technology of China Chengdu Sichuan China uestc.edu.cn

**Keywords:** discrete choice experiment, internet plus nursing service, preference, systematic review

## Abstract

**Background:**

Against the backdrop of an aging population, the traditional nursing model struggles to meet diverse needs. Despite the significant social benefits of internet plus nursing services, misaligned preferences between nurses and patients hinder their widespread adoption. Discrete choice experiments (DCEs) are a key tool for quantifying stakeholder preferences and improving service adoption. No review has systematically synthesized evidence on patients’ and nurses’ preferences for internet plus nursing services.

**Objective:**

To systematically review DCEs conducted in China that examined patients’ and nurses’ preferences for internet plus nursing services.

**Methods:**

Eight electronic databases were systematically searched from inception to September 5, 2026. Two researchers used the conjoint analysis applications in the healthcare checklist to evaluate the quality of the literature. Descriptive analysis was used to summarize the data. A hybrid deductive–inductive thematic analysis was applied to classify the attributes into broad categories and subcategories, and each attribute was analyzed for frequency, significance, relative importance, and willingness to pay. The review was registered in PROSPERO (CRD420251172405).

**Results:**

A total of 11 studies from China were included, five of which had nurses as respondents and six of which had patients as respondents. A total of 63 attributes were extracted and categorized into 7 broad categories and 25 subcategories by theme. Economic guarantee‐related attributes were the most common. Subgroup analyses showed that payment costs were the most common attribute subcategory among patients, whereas service revenue was the most common among nurses. In addition, nurses’ preferences may vary by professional title, years of experience, and income, whereas patients’ preferences may vary by income and educational attainment.

**Conclusions:**

This review identified overlapping and divergent patterns in patients’ and nurses’ preferences for internet plus nursing services. Across these studies, both groups tended to prefer greater service safety, better service accessibility, and government or public healthcare institutions as service providers. However, patient studies indicated a preference for lower payment costs, whereas nurse studies indicated a preference for higher service remuneration. Future research should further explore the preferences of diverse populations, including people from different cultural backgrounds and informal caregivers, to enhance the broader applicability of internet plus nursing services.

**Implications for Nursing Management:**

Nursing administrators should refine service plans based on preferences reported by nurses and patients regarding internet plus nursing services. These plans should address the needs of both groups while promoting the efficient, balanced allocation of home‐care nursing resources.

## 1. Introduction

As the global population ages and the prevalence of chronic diseases climbs, the traditional hospital‐centered care model is no longer able to meet the diverse and continuous health needs of the public [1]. In this context, amid ongoing social informatization, the combination of “internet plus” and medical services offers new approaches to addressing the current nursing resource shortage [2]. Internet plus nursing services refer to a model in which healthcare institutions use internet‐based technologies to arrange nursing care for discharged patients and other people with healthcare needs or mobility limitations. Nurses registered with the institutions provide services such as intravenous therapy and stoma care, primarily through an “online application and offline service delivery” model [3]. Although the model has shown significant social benefits by overcoming spatial constraints and optimizing resource allocation, its overall development remains exploratory [4]. One potential bottleneck may be the misalignment between nurses’ and patients’ preferences regarding internet plus nursing services. Therefore, it is necessary to understand the preferences of both nurses and patients for internet plus nursing services to formulate a service program that accurately addresses their needs, enhances service effectiveness, and promotes their adoption [5].

Discrete choice experiments (DCEs) are the most commonly used method for quantitatively assessing stakeholder preferences [6] and have emerged as an important tool for understanding individual preferences in health research and for guiding decision‐making in complex healthcare settings [7]. In DCEs, survey respondents are presented with hypothetical scenarios representing the attributes of a service or good and are asked to select their preferred scenario. The resulting choice data are then analyzed using choice models to determine the relative importance of different attributes in respondents’ choices. By quantifying and analyzing preferences, the results of selection data can provide references for different medical attributes [8]. DCEs can effectively identify stakeholders’ potential needs in healthcare policymaking and health technology evaluation, and are valuable for increasing the adoption of health technologies and realizing a patient‐centered service model [9, 10].

Although DCEs have been gradually applied to the field of internet plus nursing services, the results of existing studies show significant differences due to the heterogeneity of study populations and contexts. On the supply side, the attributes most valued by nurses vary, including safety and security [11], income [12], and task complexity, among others [1]. On the demand side, the attributes most valued by patients vary, including service costs [13], the nature of the organization [14], and the rate of reimbursement by health insurance [15], among others. This difference in nurse‐patient preferences, viewed through a supply‐and‐demand lens, not only limits the generalizability of a single study’s findings but also makes medical decision‐making more difficult. Therefore, there is an urgent need to synthesize existing DCEs through a systematic review and comprehensively analyze the preferences and attribute weights reported in patient and nurse studies. Such a synthesis could provide an evidence base to optimize internet plus nursing services and improve related policies.

Currently, systematic evaluations of internet plus nursing services mainly cover qualitative evidence synthesis of the nurse‐patient experience of the service [16–18] and meta‐analyses of willingness to participate and influencing factors [19, 20]. However, much of the existing evidence focuses on qualitative descriptions of the current state of services or on quantitative analyses of a single dimension. It fails to quantitatively weigh nurses’ and patients’ preferences for different service attributes in their respective decision‐making through the lens of DCEs. Moreover, because there is an imbalance between nurses’ and patients’ demands for internet plus nursing services, for example, there is a conflict of interests between nurses’ considerations of pay rewards and workload and patients’ expectations of service costs and diversified needs [5, 16, 17]. Focusing only on a single stakeholder’s perspective can easily lead to ineffective optimization measures, so the simultaneous integration of both nurse and patient preferences is critical to building sustainable service models. In addition, while one study [21] has explored stakeholder preferences for virtual care services, the scope of their services is limited to remote online digital interactions. In contrast, the internet plus nursing services of interest to this study are an “online application, offline service” model, which is fundamentally different from virtual care in terms of service security, time cost, and service content. This study aims to comprehensively evaluate nurses’ and patients’ preferences regarding the attributes of internet plus nursing services. It seeks to identify cognitive differences and demand mismatches between the two groups. This review provides evidence to support governments and healthcare institutions in developing targeted incentive policies, optimizing service delivery, and allocating resources more precisely.

## 2. Methods

This review was reported in accordance with the Preferred Reporting Items for Systematic Reviews and Meta‐Analyses (PRISMA) standards [22]. PRISMA checklist is presented in Supporting Appendix A. This study protocol was prospectively registered with PROSPERO on October 20, 2025 (CRD420251172405), before substantive literature screening and data extraction. Compared with the registered protocol, the healthcare‐professional population was limited to nurses to focus on direct providers of internet plus nursing services.

### 2.1. Data Sources and Search Strategy

Eight electronic databases were searched, including PubMed, Embase, Scopus, Cumulative Index to Nursing and Allied Health Literature (CINAHL), PsycInfo, China National Knowledge Infrastructure (CNKI), WanFang Data, and Weipu Database, and the reference lists of the included studies were manually searched. The searches covered the period from database inception to September 5, 2026, and the search terms included two main concepts: “preference” and “internet plus nursing service”. The search strategy combined medical subject headings (MeSH) and related free words. These words were identified by searching each database and referencing existing systematic reviews. A complete search strategy for all databases is provided in Supporting Appendix B.

### 2.2. Inclusion and Exclusion Criteria

Inclusion criteria were as follows: (1) study population: patients and nurses. Studies limited to community residents or the general public that did not clearly define the patient population were excluded; (2) outcome: preference for internet plus nursing services; (3) study design: observational study design with conjoint analysis, such as DCEs and best‐worst; (4) publication type: peer‐reviewed academic journal articles and dissertations; (5) language: Chinese or English. Exclusion criteria were as follows: (1) duplicate publications; (2) reviews, conference abstracts (because they often lack sufficient information for data extraction and quality assessment) [23], case reports, and other types of studies; (3) studies for which the full text was unavailable.

### 2.3. Study Identification and Selection

Two researchers (YL and HL) imported all database records into EndNote X9 for screening. After excluding duplicates, titles and abstracts were reviewed to screen potential studies that met the inclusion criteria. The full texts of potential studies were evaluated to determine the final eligible studies. If there were disagreements, they were resolved through discussions with a third researcher (YHW) until a consensus was reached.

### 2.4. Data Extraction and Data Analysis

The first researcher (YL) used a standardized Excel spreadsheet to extract the data. The extracted content included: (1) basic characteristics of the studies: first author, survey population, year of publication, country, sample size, questionnaire response rate, and research questions; (2) design features of DCEs: survey administration methods, attribute development methods, pilot testing, the total possible scenarios, number of attributes, number of choice tasks, opt‐out and status quo options, software types, and methods of analysis; (3) DCE results: attributes and their levels, statistical significance, relative importance estimates, attribute rankings, and WTP estimates with their 95% confidence intervals. A second researcher (XMX) independently verified all extracted data against the original studies and checked the subsequent conversions of the WTP estimates and their confidence intervals.

Descriptive analysis was used to summarize the studies. Given the heterogeneity in attribute naming, a hybrid deductive–inductive thematic analysis was used to classify the attributes into broad categories and subcategories for synthesis across studies. Deductively, the initial framework for broad categories drew on the five dimensions of the SERVQUAL service quality evaluation model (tangibility, assurance, reliability, empathy, and responsiveness). These dimensions reflect users’ expectations and perceptions of the service and have been widely applied in research on internet plus nursing services [2, 24, 25]. Based on the predefined SERVQUAL dimensions, we further classified the attributes according to their conceptual similarities and differences, while allowing new categories to emerge inductively during the analysis. Given that internet plus nursing services involve both patient preferences and nurses’ work preferences, the initial framework was adapted to specific contexts and expanded into seven broad categories. The first five broad categories were based on the core dimensions of SERVQUAL, while the two additional broad categories were introduced to encompass economic attributes and nurses’ work‐related attributes that were not adequately reflected in the original model. The seven broad categories are as follows: (1) service foundation guarantee, which reflects the organizational foundation for service delivery; (2) professional competence guarantee, which reflects the professional capabilities of service providers; (3) service quality guarantee, which reflects the quality and safety of services; (4) humanistic care guarantee, which reflects personalized and continuous care; (5) service efficiency guarantee, which reflects the accessibility and timeliness of services; (6) economic guarantee, which reflects economic factors related to services; and (7) work condition guarantee, which reflects work‐related conditions that affect nurses’ service delivery. Attributes with conceptually similar meanings were then inductively grouped into subcategories. To improve transparency and consistency in the classification process, the first author (YL) classified all extracted attributes, and the second researcher (XMX) independently verified all classification results. The research team then reviewed the classification results and resolved any uncertainties or disagreements through discussion until they reached consensus.

We analyzed the frequency, statistical significance, and relative importance of each attribute. The frequency of each attribute indicated how often it was included in the original DCEs; it did not reflect its importance to respondents. The overall importance of the attributes was assessed using relative importance scores and, where available, willingness‐to‐pay (WTP). For studies that did not report relative importance, relative importance was calculated using the “range method” recommended by the International Society for Pharmacoeconomics and Outcomes Research (ISPOR) [26, 27]. Descriptive subgroup analyses were conducted by respondent type (patients versus nurses).

### 2.5. Quality Assessment

The conjoint analysis applications in the healthcare checklist by ISPOR [28] were used to assess the quality of the included studies. Two researchers (YL and HL) independently assessed the quality of the included studies. In the event of disagreement, a third researcher (YHW) arbitrated the assessment. The checklist contains 10 items, which are (1) research question, (2) attributes and levels, (3) construction of tasks, (4) experimental design, (5) preference elicitation, (6) instrument design, (7) data‐collection plan, (8) statistical analyses, (9) results and conclusions, and (10) study presentation. Each item consists of three subitems. Subitems meeting the relevant criteria were rated “Yes”; those that did not meet the criteria or for which the relevant information was not reported were rated “No.” Checklist adherence was reported descriptively. Because too few independent studies reported comparable effect estimates for the same attribute, funnel plots and Egger’s regression test were not applicable. Therefore, we did not formally assess publication bias.

## 3. Results

### 3.1. Literature Search

This search yielded 1408 studies, with 456 duplicates removed, 779 excluded after reading the title and abstract, and 162 excluded after reading the full text. In the end, 11 studies were included in this study. The PRISMA flow chart for the study screening process is depicted in Figure 1.

**FIGURE 1:**
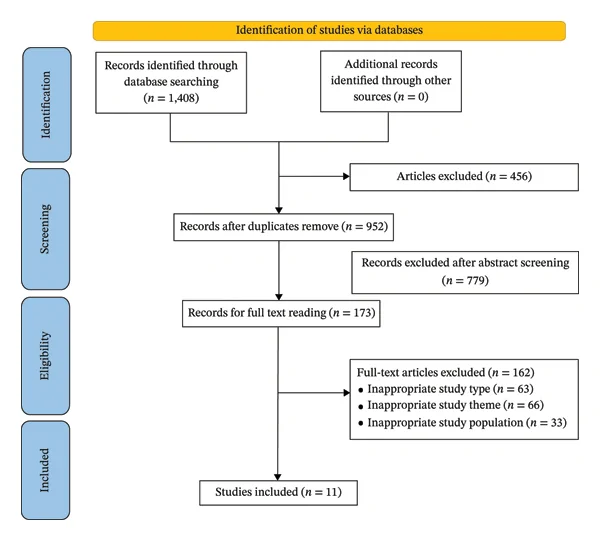
PRISMA flow chart for the study screening process.

### 3.2. Study Characteristics

A total of 11 studies were included in this study, published between 2023 and 2025. Five studies [1, 11, 12, 29, 30] had nurses as respondents. Six studies [13–15, 31–33] had patients as respondents, and patient categories included cancer patients, disabled elderly people, older patients with chronic diseases, and patients with acute mastitis and hypogalactia. Sample sizes ranged from 105 to 770 participants, with 3533 participants overall. Respondents’ response rates ranged from 74.85% to 100%. Three studies [11, 12, 30] used online questionnaire collection, seven studies [13–15, 29, 31–33] used face‐to‐face questionnaire collection, and one study [1] used a combination of online and face‐to‐face questionnaire collection.

All studies reported methods for attribute development, and seven studies [1, 12, 15, 29, 30, 32, 33] used a combination of three attribute development methods: literature review, qualitative research, and expert consultation. One study [32] was unclear as to whether a pilot test was performed. Four studies [1, 11, 12, 33] reported the total number of possible choice scenarios, ranging from 108 to 864. The number of attributes is set to 5 to 7, and the number of choice tasks ranges from 6 to 36. All studies were DCEs. Experimental designs for DCEs were created in SPSS (*n* = 2), Sawtooth (*n* = 1), Stata (*n* = 4), SAS (*n* = 2), or Ngene (*n* = 2) software. Most of the studies were statistically analyzed using mixed logit models and Stata software, as shown in Tables 1 and 2.

**TABLE 1:** Basic characteristics of the included studies.

Author (year)	Respondent type	Patient group	Sample size	Response rate (%)	Research question
Du et al. (2025) [31]	Patient	Colorectal cancer patients	189	82.54	Preferences of colorectal cancer patients for “internet + TCM nursing services”
He et al. (2024) [12]	Nurse	—	220	95.24	Sharing nurses’ preferences for job attributes
Liu et al. (2024) [14]	Patient	Older adults with disabilities	253	74.85	Preferences of disabled elderly people for home‐based medical care services
Liu et al. (2024) [15]	Patient	Older patients	395	96.30	Preferences of elderly patients for “internet + nursing service”
Liu et al. (2025) [29]	Nurse	—	770	90.38	Preferences of nurses in public hospitals for providing “internet plus nursing services”
Liu et al. (2025) [32]	Patient	Elderly patients with chronic diseases	342	100%	Preferences of elderly chronic disease patients for “internet + nursing services”
Mu et al. (2024) [13]	Patient	Patients with acute mastitis and hypogalactia	105	92.90	Preferences of patients for internet‐based sharing nursing services for breast massage
Shui et al. (2023) [30]	Nurse	—	420	80.50	Nurses’ preference for “internet + nursing service” for older patients
Wang (2025) [1]	Nurse	—	372	90.51	Nurses’ job preferences in tertiary public hospitals regarding “internet plus nursing service”
Yang et al. (2025) [11]	Nurse	—	211	100%	Registered nurses’ preferences for Uber‐style internet plus nursing services
Zhang (2025) [33]	Patient	Patients with advanced cancer	256	95.52	Advanced cancer patients’ preferences for home hospice care

*Note:* “—”, not reported. The two studies reporting a 100% response rate administered questionnaires onsite to eligible participants under investigator supervision.

Abbreviation: TCM, Traditional Chinese Medicine.

**TABLE 2:** DCE characteristics of the included studies.

Author (year)	Survey administration method	Attribute development	Piloting	Total possible scenarios	No. of attributes	No. of choice tasks	Opt‐out/status quo option	Experimental design software	Methods of analysis	Data analysis software
Du et al. (2025) [31]	Self‐completed (offline)	ac	Yes	—	6	6	No	SPSS	Mixed logit models	Stata
He et al. (2024) [12]	Self‐completed (online)	abc	Yes	864	6	12	Yes	Sawtooth	Multinomial logit model	—
Liu et al. (2024) [14]	Self‐completed (offline)	ac	Yes	—	6	12	No	SAS	Mixed logit models	Stata
Liu et al. (2024) [15]	Self‐completed (offline)	abc	Yes	—	6	9	Yes	Stata	Conditional logit model	Stata
Liu et al. (2025) [29]	Self‐completed (offline)	abc	Yes	—	5	9	Yes	Stata	Mixed logit models	Stata
Liu et al. (2025) [32]	Self‐completed (offline)	abc	—	—	6	8	Yes	Stata	Mixed logit models	Stata
Mu et al. (2024) [13]	Self‐completed (offline)	ac	Yes	—	7	36	No	Ngene	Conditional logit model and mixed logit model	Stata
Shui et al. (2023) [30]	Self‐completed (online)	abc	Yes	—	5	8	No	SAS	Mixed logit models	Stata
Wang (2025) [1]	Self‐completed (online/offline)	abc	Yes	486	6	12	No	Ngene	Mixed logit models	Stata
Yang et al. (2025) [11]	Self‐completed (online)	ac	Yes	108	5	7‐8	No	SPSS	Mixed logit models	Stata
Zhang (2025) [33]	Self‐completed (offline)	abc	Yes	324	5	9	No	Stata	Conditional logit model and mixed logit model	Stata

*Note:* “—”, not reported; a, literature review; b, interviews; c, expert consultation.

### 3.3. Quality Assessment

The 11 studies met 24–30 of the 30 subitems on the ISPOR checklist, with adherence varying across assessment domains. All studies clearly stated their research questions. The selection of attributes and levels for all studies was supported by the literature review. Seven studies [1, 12, 15, 29, 30, 32, 33] also used qualitative methods to develop attributes. Only four studies [12, 15, 29, 32] included an opt‐out or status quo option. All studies collected appropriate respondent information, and only one study [29] did not define the attribute. Most of the studies used appropriate data collection methods and statistical analyses, while only one study [33] did not describe the sampling method, and three [14, 29, 30] did not mention ethical considerations. The majority of the studies were reported in full, and the results were fully discussed; only two studies [14, 30] did not adequately discuss the limitations of the study, as shown in Supporting Table 1 in Supporting Appendix C.

### 3.4. Classification of the Attributes

In this study, a total of 63 attributes were extracted from 11 studies, and a summary of all attribute classifications is shown in Table 3. The 63 attributes were categorized as: economic guarantee, 14 items (22%); service quality guarantee, 12 items (19%); service efficiency guarantee, 10 items (16%); humanistic care guarantee, 9 items (14%); work condition guarantee, 7 items (11%); service foundation guarantee, 6 items (10%); and professional competence guarantee, 5 items (8%); see Figure 2A. Of the 25 subcategories, payment costs and service accessibility were the most common (*n* = 6, 10% for each), followed by professional qualifications, service revenue, and service safety (*n* = 5, 8% for each). Other common subcategories include service content (*n* = 4.6%), service provider (*n* = 4.6%), and individualized service (*n* = 4.6%); see Figure 2B.

**TABLE 3:** Classification mapping of attributes across the included studies.

Author (year)	Attribute wording (direct from study)	Broad category	Subcategory
Du et al. (2025) [31]	Payment costs	Economic guarantee	Payment costs
	Nurse qualification	Professional competence guarantee	Professional qualifications
	Service approach	Work condition guarantee	Working mode
	Service wait time	Service efficiency guarantee	Service accessibility
	Service comprehensiveness	Service quality guarantee	Service content
	Evaluation of platform services	Service quality guarantee	User ratings

He et al. (2024) [12]	Working modes	Work condition guarantee	Working mode
	Duration per visit	Service efficiency guarantee	Service duration
	Income per visit	Economic guarantee	Service revenue
	Personal safety management	Service quality guarantee	Service safety
	Medical risk prevention	Service quality guarantee	Service safety
	Refresher training	Work condition guarantee	Continuing education

Liu et al. (2024) [14]	Reservation method	Service efficiency guarantee	Reservation method
	Service content	Service quality guarantee	Service content
	Service decision‐making	Humanistic care guarantee	Subjects of decision
	Nurses’ experience	Professional competence guarantee	Professional qualifications
	Character of structure	Service foundation guarantee	Service provider
	Payment costs	Economic guarantee	Payment costs

Liu et al. (2024) [15]	Single service cost	Economic guarantee	Payment costs
	Hospital size	Service foundation guarantee	Organization size
	Service content	Humanistic care guarantee	Individualized service
	Service provider	Professional competence guarantee	Professional qualifications
	Medical insurance reimbursement rate	Economic guarantee	Reimbursement ratio
	Service platform	Service foundation guarantee	Service provider

Liu et al. (2025) [29]	Service costs	Economic guarantee	Service revenue
	Incentive mechanism	Work condition guarantee	Organizational incentives
	Service mode	Work condition guarantee	Working mode
	Service content	Humanistic care guarantee	Individualized service
	Service platform	Service foundation guarantee	Service provider

Liu et al. (2025) [32]	Service costs	Economic guarantee	Payment costs
	Service content	Humanistic care guarantee	Individualized service
	Service platform	Service foundation guarantee	Service provider
	Hospital size	Service foundation guarantee	Organization size
	Reimbursement ratio	Economic guarantee	Reimbursement ratio
	Service provider	Professional competence guarantee	Professional qualifications

Mu et al. (2024) [13]	Service costs	Economic guarantee	Payment costs
	Nurse qualification	Professional competence guarantee	Professional qualifications
	Response time	Service efficiency guarantee	Service accessibility
	Evaluation of platform services	Service quality guarantee	User ratings
	Health guidance and follow‐up	Humanistic care guarantee	Continuing care
	Service safety	Service quality guarantee	Service safety
	Strength of hospital supervision	Service quality guarantee	Service supervision

Shui et al. (2023) [30]	Service revenue	Economic guarantee	Service revenue
	Service content	Service quality guarantee	Service content
	Service distance	Service efficiency guarantee	Service accessibility
	Service continuity	Humanistic care guarantee	Continuing care
	Living status	Humanistic care guarantee	Living arrangement

Wang (2025) [1]	Business consideration	Economic guarantee	Service revenue
	Service distance	Service efficiency guarantee	Service accessibility
	Service time	Service efficiency guarantee	Service time slots
	Task complexity	Work condition guarantee	Task complexity
	Nursing operation hours	Service efficiency guarantee	Service duration
	Service safety	Service quality guarantee	Service safety

Yang et al. (2025) [11]	Income	Economic guarantee	Service revenue
	Patient and family cooperation	Work condition guarantee	Patient and family cooperation
	Safety and security	Service quality guarantee	Service safety
	Commute time	Service efficiency guarantee	Service accessibility
	Service type	Service quality guarantee	Service content

Zhang (2025) [33]	Source of payment	Economic guarantee	Payment method
	Service content	Humanistic care guarantee	Individualized service
	Service costs	Economic guarantee	Payment costs
	Quality of life	Humanistic care guarantee	Quality of life
	Waiting period for postbooking care	Service efficiency guarantee	Service accessibility

**FIGURE 2:**
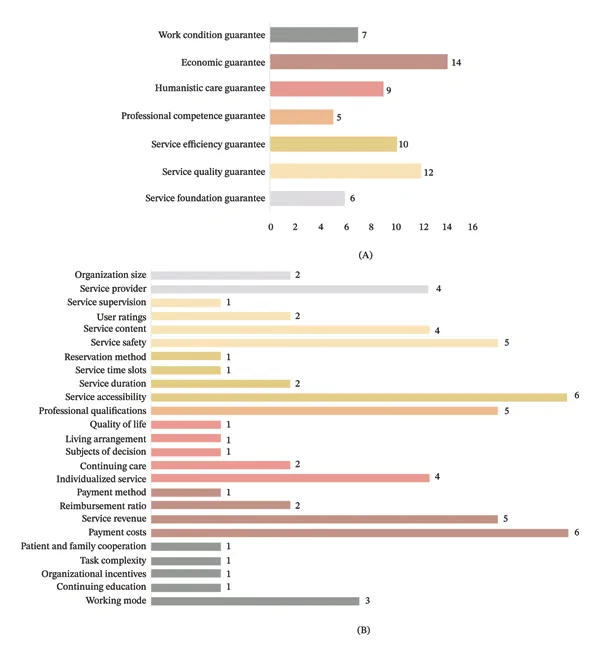
(A). Frequency of attribute broad categories across all included studies. (B). Frequency of attribute subcategories across all included studies.

The frequency of individual attributes varied depending on the respondent’s perspective; therefore, this study summarized the proportion of subcategories of attributes from the patient’s and nurse’s perspectives, respectively, as shown in Table 4. In studies conducted from the patient perspective, payment costs (17%) and professional qualifications (14%) were the most common subcategories. In contrast, service revenue (19%), service safety (15%), and service accessibility (11%) were more common in studies conducted from the nurses’ perspective.

**TABLE 4:** Proportion of attribute preference estimates across subcategories and respondent perspective.

Attribute subcategory	Overall (*n* = 63)	Respondent perspective	
		Patient (*n* = 36)	Nurse (*n* = 27)
Payment costs	6 (10%)	6 (17%)	0 (0%)
Service accessibility	6 (10%)	3 (8%)	3 (11%)
Professional qualifications	5 (8%)	5 (14%)	0 (0%)
Service revenue	5 (8%)	0 (0%)	5 (19%)
Service safety	5 (8%)	1 (3%)	4 (15%)
Service provider	4 (6%)	3 (8%)	1 (4%)
Service content	4 (6%)	2 (6%)	2 (7%)
Individualized service	4 (6%)	3 (8%)	1 (4%)
Working mode	3 (5%)	1 (3%)	2 (7%)
Service duration	2 (3%)	0 (0%)	2 (7%)
User ratings	2 (3%)	2 (6%)	0 (0%)
Organization size	2 (3%)	2 (6%)	0 (0%)
Reimbursement ratio	2 (3%)	2 (6%)	0 (0%)
Continuing care	2 (3%)	1 (3%)	1 (4%)
Task complexity	1 (2%)	0 (0%)	1 (4%)
Quality of life	1 (2%)	1 (3%)	0 (0%)
Organizational incentives	1 (2%)	0 (0%)	1 (4%)
Payment method	1 (2%)	1 (3%)	0 (0%)
Reservation method	1 (2%)	1 (3%)	0 (0%)
Living arrangement	1 (2%)	0 (0%)	1 (4%)
Patient and family cooperation	1 (2%)	0 (0%)	1 (4%)
Continuing education	1 (2%)	0 (0%)	1 (4%)
Subjects of decision	1 (2%)	1 (3%)	0 (0%)
Service supervision	1 (2%)	1 (3%)	0 (0%)
Service time slots	1 (2%)	0 (0%)	1 (4%)

### 3.5. Significance of the Attributes

All studies reported the significance of the attributes, with 55 attributes (87%) being statistically significant, indicating that different levels of these attributes had an impact on respondents’ choice behavior. Eight attributes (13%) were not significant. All attributes classified under economic guarantee, service quality guarantee, and service foundation guarantee were statistically significant; see Table 5.

**TABLE 5:** Attributes, levels, and statistical significance of the included studies.

Author (year)	Attribute wording (direct from study)	Statistically significant	No. of levels	Level range (direct from study)
Du et al. (2025) [31]	Payment costs	Yes	3	50 Yuan; 100 Yuan; 150 Yuan
	Nurse qualification	Yes	2	General nurse; Chinese medicine nurse specialist
	Service approach	No	3	Remote service; door‐to‐door service; remote service + door‐to‐door service
	Service wait time	No	2	30; 60 min
	Service comprehensiveness	Yes	3	General; comprehensive; very comprehensive
	Evaluation of platform services	Yes	3	Ordinary; good; excellent

He et al. (2024) [12]	Working modes	No	2	Part‐time work; full‐time work
	Duration per visit	Yes	4	1; 2; 3; 4 h
	Income per visit	Yes	4	100 Yuan; 250 Yuan; 400 Yuan; 550 Yuan
	Personal safety management	Yes	3	Superior; medium; basic
	Medical risk prevention	Yes	3	Superior; medium; basic
	Refresher training	No	3	Less than 4; 4–8 h; more than 8 h

Liu et al. (2024) [14]	Reservation method	Yes	3	Offline reservation; online reservation; both online and offline
	Service content	Yes	3	Basic care package; specialty care packages; health management packages
	Service decision‐making	No	3	Older persons mainly; informal caregivers mainly; service provider mainly
	Nurses’ experience	No	2	3 years; 5 years
	Character of structure	Yes	2	Public institutions; nonpublic institutions
	Payment costs	Yes	3	100 Yuan; 200 Yuan; 300 Yuan

Liu et al. (2024) [15]	Single service cost	Yes	3	150 Yuan; 250 Yuan; 350 Yuan
	Hospital size	Yes	3	Large; medium; smaller
	Service content	No	2	Basic services; individual services
	Service provider	Yes	3	Junior nurse; intermediate nurse; advanced practice nurse
	Medical insurance reimbursement rate	Yes	3	30%; 50%; 70%
	Service Platform	Yes	3	Government‐led; hospital‐led; enterprise‐led

Liu et al. (2025) [29]	Service costs	Yes	3	150 Yuan; 250 Yuan; 350 Yuan
	Incentive mechanism	Yes	4	None; integration into performance appraisal; priority in title promotion; priority given to further education and training
	Service mode	Yes	2	Individual home visits; team home service
	Service content	Yes	2	Basic package; personalized package
	Service platform	Yes	2	Business‐led; government‐led

Liu et al. (2025) [32]	Service costs	Yes	3	150 Yuan; 250 Yuan; 350 Yuan
	Service content	Yes	2	Basic package; personalized package
	Service platform	Yes	3	Business‐led; government‐led; hospital‐led
	Hospital size	Yes	3	Small; medium; large
	Reimbursement ratio	Yes	3	30%; 50%; 70%
	Service provider	Yes	3	Primary nurse; intermediate nurse; advanced nurse

Mu et al. (2024) [13]	Service costs	Yes	4	200 Yuan; 300 Yuan; 400 Yuan; 500 Yuan
	Nurse qualification	Yes	3	2 years–5 years; 6 years–10 years; > 10 years
	Response time	Yes	3	30 min; 60 min; 90 min
	Evaluation of platform services	Yes	3	Average; good; very good
	Health guidance and follow‐up	Yes	2	Provide; not available
	Service safety	Yes	3	Buying accident insurance; equipped with care recorders; double‐person door‐to‐door service
	Strength of hospital supervision	Yes	2	Small; large

Shui et al. (2023) [30]	Service revenue	Yes	4	50 Yuan; 100 Yuan; 150 Yuan; 200 Yuan
	Service content	Yes	3	Specialized nursing; general nursing; health promotion
	Service distance	Yes	3	< 5; 5–10 km; 11–15 km
	Service continuity	Yes	3	None; low; high
	Living status	Yes	3	Living with family; live alone; residence in a nursing home

Wang (2025) [1]	Business consideration	Yes	3	80 Yuan; 100 Yuan; 120 Yuan
	Service distance	Yes	3	< 3; 3–6 km; > 6 km
	Service time	No	2	Nurses’ rest day mornings; nurses’ rest day afternoons
	Task complexity	Yes	3	Easy; medium; difficult
	Nursing operation hours	Yes	3	< 15 min; 15 min–30 min; > 30 min
	Service safety	Yes	3	Good; medium; normal

Yang et al. (2025) [11]	Income	Yes	3	Current income; 15% increase in current income; 30% increase in current income
	Patient and family cooperation	Yes	3	Poor cooperation; moderate cooperation; good cooperation
	Safety and security	Yes	3	Low security; medium security; high security
	Commute time	Yes	2	> 30 min; ≤ 30 min
	Service type	Yes	2	Invasive operations; noninvasive operations

Zhang (2025) [33]	Source of payment	Yes	4	Self‐paying; medical insurance; commercial insurance; other sources
	Service content	Yes	3	Basic services + hospice preparation + grief counseling; basic services + life (death) education + advance living wills; basic services + grief counseling + family care support
	Service costs	Yes	3	100 Yuan; 200 Yuan; 300 Yuan
	Quality of life	Yes	3	Good; general; bad
	Waiting period for postbooking care	Yes	3	6–12 h; 13–24 h; 25–48 h

### 3.6. Relative Importance of the Attributes

The relative importance rankings of the attributes varied significantly between studies, with a total of 8 most important attribute subcategories (ranked first); see Figure 3. Among the 11 included studies, payment costs ranked first in three studies (27%), while service revenue ranked first in two studies (18%). The relative importance scores for each attribute are shown in Supporting Table 2. A complete list of attribute subcategory ranking frequencies can be found in Supporting Table 3.

**FIGURE 3:**
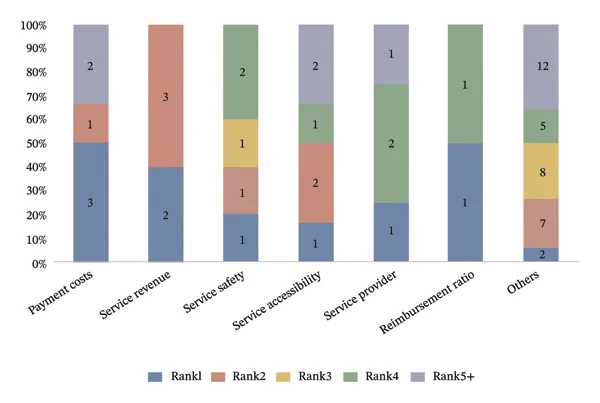
Frequency of attribute importance ranks by attribute subcategory (top six subcategories).

The subgroup analysis reveals that from the patient perspective, payment costs are the most important attribute subcategory, followed by service provider, reimbursement ratio, and quality of life. From the nurses’ perspective, service revenue is the most important attribute subcategory, followed by service safety, service accessibility, and task complexity. For subgroup analyses of the order of the frequencies of importance ranks for attribute subcategories, see Supporting Tables 4 and 5 and Supporting Figure 1.

### 3.7. Preference Interactions

Four studies assessed how different participant characteristics affected preferences for internet plus nursing services. For nurses, groups of nurses with senior professional titles, more experience, and higher incomes placed more emphasis on service safety. In contrast, junior, less experienced, and low‐income nurses were more concerned with nursing task complexity and service distance [1, 11]. For patients, high‐income, highly educated patients preferred services provided by public institutions, and less‐educated patients valued service content comprehensiveness and platform service ratings more [14, 31]; see Supporting Table 6.

### 3.8. WTP

WTP estimates and corresponding 95% confidence intervals were standardized to the 2025 price level in Chinese yuan (CNY) using the Consumer Price Index (CPI) reported by the National Bureau of Statistics of China, based on each study’s data collection year. The adjusted WTP estimates are summarized in Supporting Table 7. Ten studies (91%) reported WTP estimates. Among patients, higher WTP estimates were reported for more favorable levels of platform service evaluation, quality of life, insurance reimbursement, nursing qualifications, and hospital size. Among nurses, higher WTP estimates were reported for more favorable levels of safety and security, patient and family cooperation, commute time, service type, and incentive mechanism.

## 4. Discussion

To our knowledge, this is the first review to synthesize preference evidence on internet plus nursing services from both patient and nurse perspectives. Across separate DCEs, patient and nurse studies showed similar preference patterns for greater service safety, better service accessibility, and government or public healthcare institutions as service providers. Their economic priorities, however, differed: patient studies emphasized lower payment costs, whereas nurse studies emphasized higher service remuneration. These findings may indicate a potential misalignment between service affordability and provider remuneration. Further analysis of preference heterogeneity showed that nurses’ preferences varied by professional title, years of experience, and income. In contrast, patients’ preferences varied by income and educational attainment, highlighting differences in service preferences among populations with different characteristics. This study identifies factors relevant to designing internet plus nursing service models and informing policy, providing evidence to optimize service delivery while considering the priorities of both groups.

Overall, the available reports of DCEs applied to patients’ and nurses’ preferences for internet plus nursing services are more complete, but there are still some areas for improvement. Most studies relied on literature reviews to develop attributes, and only seven studies [1, 12, 15, 29, 30, 32, 33] used qualitative methods. This limited use of qualitative methods may have increased the risk that relevant attributes or levels were omitted. Future research should use a combination of literature review, qualitative research, and expert consultation to improve predictive validity and reduce bias [34]. Some of the studies used ranges to define attribute levels rather than the type of levels recommended by ISPOR. This vague definition tends to trigger a level of subjective interpretation by the respondents, and the resulting ambiguity can affect the results [28]. ISPOR’s inventory notes that interviewer‐led survey administration improves the quality of the data because the interviewer can explain the task and answer the questions more fully [28]. However, only seven studies [13–15, 29, 31–33] utilized face‐to‐face surveys, and the remaining studies mainly utilized self‐directed online completion, which may have resulted in an inadequate understanding of the DCE selection task by some participants. Future research should be based on the ISPOR checklist, strengthening the application of qualitative methods in the attribute development stage, adopting appropriate attribute level setting methods, and selecting survey methods according to the characteristics of the target group, in order to enhance the methodological rigor and reliability of the results of the study. In addition, some included studies reported relatively wide confidence intervals for WTP estimates, indicating uncertainty in the original DCE estimates, which may partly reflect preference heterogeneity among respondents. This uncertainty should be considered when interpreting WTP estimates across studies.

Attributes related to economic guarantee are the most commonly included attributes in original DCE studies. It should be noted that attribute frequency reflects the inclusion of attributes in the original study, rather than their importance to respondents. In terms of preferences, patients and nurses have different priorities. For patients, payment costs are the most important, which is similar to the results of other studies [35, 36] showing that payment costs directly influence patients’ preference for service selection. However, the cost of internet plus nursing services is about 5–20 times higher than the cost of the same service in a brick‐and‐mortar hospital [37], as the fee for in‐home services is mainly based on the pricing of local healthcare services and overlaid with the cost of transportation and labor. In addition, subgroup analysis further reveals that the reimbursement ratio is the second most important subcategory of economic guarantee in patients’ perceptions. However, at present, the cost of services in most regions is still dominated by patients’ out‐of‐pocket expenses, which have not yet been fully incorporated into the scope of medical insurance, and the price regulation mechanism is imperfect [38, 39], limiting the accessibility and promotion of services [32]. In the future, there is a need to accelerate the inclusion of the cost of internet plus nursing services into the health insurance reimbursement system and improve the reimbursement ratio, as well as to strengthen price regulation. In addition, reference can be made to the pilot experience of long‐term care insurance, and commercial insurance can be used as a supplement to medical insurance to build a diversified payment system and reduce the financial burden on patients.

For nurses, service revenue is most important, and other studies have shown that increasing financial income is the greatest motivation for nurses to engage in multiple‐site practice [40, 41]. Analyzing the reasons, nurses participating in internet plus nursing services need to invest additional time and effort and face additional risks, such as personal safety concerns and medical disputes in home‐based service scenarios. The mismatch between service remuneration and the associated risks and workload may affect nurses’ preferences [30]. In addition to the general concern about service revenue, subgroup analyses show that the senior nurse group shows greater sensitivity to service safety, which may be related to senior nurses’ perceptions of the need for self‐protection [42]. Junior nurses are more concerned with task complexity and service distance. Therefore, under the premise of guaranteeing safety, the platform should implement accurate matching when dispatching orders, matching senior nurses with specialty projects with a certain degree of difficulty, and assigning closer and more basic nursing operations to junior nurses, to maximize the allocation efficiency of nursing resources. In addition, a dynamic adjustment mechanism linking service prices to service difficulty, service quality, safety risks, and patient evaluation scores should be established in the future [43]. Specifically, for high‐risk or more complex procedures, a certain percentage may be added to the base service fee, and a portion of that additional fee may be paid to nurses as a risk allowance.

In service quality guarantee, service safety is the most valued attribute category for nurses, and higher levels of safety are preferred. Studies have shown that many healthcare workers have experienced workplace violence in home care, which affects their physical and mental health and even leads to resignation [44]. In addition to nurse safety, patient safety in home care is equally important. Studies on vulnerable populations have emphasized the importance of individualized medication management and monitoring for adverse reactions [45], and have pointed out that age and polypharmacy may increase the associated risks [46]. Related research has further proposed safety management strategies such as electronic health records, personalized medication management, patient education, and multidisciplinary teams [47]. Another study has shown that machine learning can predict drug‐related side effects, providing a basis for developing a more systematic home safety management model for internet plus nursing services [48]. Regarding service efficiency guarantees, service accessibility is a subcategory of attributes frequently included in studies involving patients and nurses. Overall, existing research indicates that patients tend to prefer shorter wait times, while nurses prefer services with shorter travel distances. Especially in advanced cancer patients, who are highly sensitive to the cost of time, service accessibility can directly affect their perceived quality of life [33]. From a holistic perspective on digital nursing services, both virtual nursing services [21] and internet plus nursing services focus on professional qualifications, service fees, and reimbursement. However, the latter also involves factors such as service accessibility, home safety, and service supervision, suggesting that as services shift from remote interactions to in‐home visits, preferences may be further influenced by the service context.

In humanistic care guarantee, individualized service is the most frequently included attribute subcategory in patient studies. Although individualized service is regarded as an effective means of bringing about behavioral change in patients [49], some studies suggest that patients tend to opt for basic service packages. This preference may be related to higher medical costs, inadequate health insurance coverage, and a lack of awareness [15]. Therefore, personalized service design should consider patients’ actual needs and financial burdens to increase acceptance of these services. In terms of work condition guarantees, nurses prefer part‐time work and team‐based home visits, reflecting their concerns for job stability and safety during home visits. At the same time, task complexity may increase the stress nurses experience when providing care in a home setting without the support of an in‐hospital team or emergency backup [18]. Incentives such as performance evaluations, professional title promotions, and continuing education reflect nurses’ career development needs. Moreover, nurses show a clear preference for good patient and family cooperation. Previous research has also demonstrated that high‐quality physician‐patient interactions contribute to the functioning and well‐being of the entire family [50], thereby facilitating the management of disease states [51].

Regarding the service foundation guarantee, patients and nurses generally prefer government or public healthcare institutions as service providers. This may be related to the limitations of businesses or nonpublic institutions in terms of service quality and public trust [52, 53]. Therefore, brick‐and‐mortar healthcare institutions should be encouraged to participate in and help regulate the management of corporate platforms, thereby promoting the standardized development of diverse service providers [32]. In the professional competence guarantee, professional qualifications are a key factor in patients’ perceptions of quality, and nurses with specialty qualifications or advanced practice nurse status are associated with greater patient willingness to pay. This may stem from the specialized strengths of high‐level nurses in complex case management and emergency coverage, allowing them to more accurately match individualized needs in home care [32].

Because all included evidence came from China, the findings should be interpreted with caution. In China, internet plus nursing services are still primarily paid for out of pocket, and regional differences in health insurance policies exist [37]. Meanwhile, some countries have already established mechanisms, such as long‐term care insurance, to cover the costs of home care [43, 54]. Therefore, different health insurance systems may influence patients’ preferences regarding reimbursement rates. In addition, since China launched the internet plus nursing services pilot program in 2019, these services have primarily been provided through brick‐and‐mortar medical institutions [3]. In China, the dominant role of public healthcare institutions and the public’s trust in them may also influence patients’ and nurses’ choices of service providers [55, 56]. This differs from home care service models in some countries, where third‐party organizations or healthcare companies lead services [54]. In contrast, some of the findings may have broader applicability. For example, the differing economic preferences reported across patient and nurse studies may reflect a potential trade‐off between reducing patients’ financial burden and ensuring fair compensation for nurses’ work. In addition, the emphasis on service safety, accessibility, and professional qualifications may also reflect the fundamental needs for safety, convenience, and professional competence in home care services. However, these findings still need further validation across different healthcare systems and cultural contexts.

### 4.1. Implications for Future Research

By systematically integrating evidence on the preferences of both supply and demand, this study reveals the key attributes affecting the preferences of both parties and provides a scientific, evidence‐based basis for constructing an accurately adapted internet plus nursing services system. Future studies should further broaden the depth and breadth of attribute development, explore the impact of internal attributes, such as emotional support, on preferences, and further investigate differences in preferences among informal caregivers and people with different diseases. Meanwhile, the scope of the study should be extended to rural and remote areas where medical resources are relatively scarce, and the study should be conducted in areas with different cultural backgrounds, so as to enhance the equity and universality of internet plus nursing services.

### 4.2. Implications for Nursing Management

The findings of this review can inform the management of internet plus nursing services. For safety governance, policymakers, industry regulators, and nursing administrators should improve the systems for certifying nurses to provide in‐home services and for assessing risks in home environments. At the same time, the relevant regulatory authorities should establish a two‐way credit assessment mechanism, supplemented by professional liability insurance, a one‐button emergency alarm system, and emergency response measures. For personnel management, medical institutions and service platforms should assign appropriately qualified nurses based on patient needs and task complexity. Service distance should also be factored into intelligent scheduling, and flexible scheduling should be used to reduce nurses’ travel burden and workloads. For payment, efforts should focus on covering these services through medical insurance or long‐term care insurance and gradually developing diversified payment mechanisms. Nurses’ compensation should reflect the difficulty of the work, the level of risk, the quality of care, and the time required. Medical institutions should also strengthen training in family‐centered care to enhance nurses’ safety management and emergency response capabilities. Participation in internet plus nursing services can be linked to performance evaluations, professional advancement, and continuing education, supporting nurses’ professional development. In addition, hospitals, communities, and service platforms should coordinate home‐care nursing resources to improve access to services in underserved areas.

### 4.3. Limitations

The present study has the following limitations: first, only studies published in English and Chinese were included, which may introduce language bias. Second, the included studies were all from China, and, given the specific healthcare system and culture, the generalizability of the results to other regions is limited; regional adjustments are needed. Third, attribute classification involved some subjective judgment, and alternative classification schemes may be possible. Finally, patients were not involved in the design or conduct of this review; future studies may benefit from patient involvement.

## 5. Conclusion

Evidence synthesized from separate DCEs showed both overlapping and differing preference patterns between patient and nurse studies. Studies of both groups generally reported preferences for greater service safety, better service accessibility, and government or public healthcare institutions as service providers. However, patient studies emphasized lower payment costs, whereas nurse studies emphasized higher service remuneration, suggesting a potential misalignment between service affordability and provider remuneration. Meanwhile, attributes such as professional qualifications, task complexity, and family cooperation influenced the preferences of both parties. The differing preference patterns reported across patient and nurse studies highlight the need for future platforms to incorporate safety safeguards, diversified payment mechanisms, and needs‐based scheduling. Further exploration of preferences across different populations, including cross‐cultural backgrounds and informal caregivers, is needed to improve the broader applicability of internet plus nursing services.

## Author Contributions

Conceptualization, writing–original draft and methodology: Yue Li, Xiujuan Zhang, Yingxin Li, and Xuemei Xie; data curation and formal analysis: Yue Li, Wenqian Su, Xi Huang, and Hang Li, Xuemei Xie; writing–review and editing, supervision: Xiujuan Zhang and Yaohua Wu. All authors contributed to the manuscript revision.

## Funding

This study was funded by the Nursing Department of West China Second University Hospital, Sichuan University (HLB‐J‐202601).

## Disclosure

All authors read and approved the submitted version. The funding source did not participate in the design of this study, the collection and analysis of data, the writing of the manuscript, or decisions regarding its publication.

## Ethics Statement

As this study is a systematic review of previously published literature, it did not involve direct contact with human participants or the collection of identifiable personal data. Therefore, institutional review board (IRB) approval was not required.

## Conflicts of Interest

The authors declare no conflicts of interest.

## Supporting Information

Additional supporting information can be found online in the Supporting Information section.

## Supporting information


**Supporting Information** The PRISMA Checklist is available in Supporting Appendix A. All database search strategies are presented in Supporting Appendix B. Supporting tables and figures are available in Supporting Appendix C.

## Data Availability

The data that support the findings of this study are available in the supporting information of this article.
