# Supplementary material for: Preferences for Internet Plus Nursing Services Among Nurses and Patients in China: A Systematic Review of Discrete Choice Experiments
Source: J Nurs Manag. 2026 Oct 4;2026:8852180. doi: 10.1155/jonm/8852180 (PMC13635678; doi:10.1155/jonm/8852180)
Supplement: Supplementary file 1 [file JONM-2026-8852180-s001.zip › Supplemental Appendix C Tables and figures.docx]

**Supplemental Appendix C: Supplementary Tables and figures**

[Supplemental Table 1. Evaluation results of literature quality	2](#_Toc28225)

[Supplemental Table 2. The classification of attributes included in the study and their relative importance and ranking	4](#_Toc4576)

[Supplemental Table 3. Frequencies of attribute importance ranks by attribute type (full list)	7](#_Toc2647)

[Supplemental Table 4. Frequencies of attribute importance ranks by attribute type (patient's perspective)	9](#_Toc1447)

[Supplemental Table 5. Frequencies of attribute importance ranks by attribute type (nurse's perspective)	10](#_Toc31115)

[Supplemental Table 6. Summary of preference heterogeneity reported in the included studies	11](#_Toc28822)

[Supplemental Table 7. Marginal willingness to pay estimates	12](#_Toc18114)

[Supplemental Figure 1 (A)	17](#_Toc14341)

[Supplemental Figure 1 (B)	18](#_Toc22487)

| Supplemental Table 1. Evaluation results of literature quality | | | | | | | | | | | | | | | | | | | | | | | | | | | | | | |
| --- | --- | --- | --- | --- | --- | --- | --- | --- | --- | --- | --- | --- | --- | --- | --- | --- | --- | --- | --- | --- | --- | --- | --- | --- | --- | --- | --- | --- | --- | --- |
| Studies | Items | | | | | | | | | | | | | | | | | | | | | | | | | | | | | |
|  | 1.Well-defined research question | | | 2. Attributes and levels of choice supported by evidence | | | 3. Appropriate construction of tasks | | | 4. Experimental design justified and evaluated | | | 5. Appropriate Preferences elicit | | | 6. Appropriate design of data collection instrument | | | 7. Appropriate data collection plan | | | 8. Appropriate statistical analyses | | | 9. Valid results and conclusions | | | 10. Clear, concise, and complete study presentation | | |
|  | 1.1 | 1.2 | 1.3 | 2.1 | 2.2 | 2.3 | 3.1 | 3.2 | 3.3 | 4.1 | 4.2 | 4.3 | 5.1 | 5.2 | 5.3 | 6.1 | 6.2 | 6.3 | 7.1 | 7.2 | 7.3 | 8.1 | 8.2 | 8.3 | 9.1 | 9.2 | 9.3 | 10.1 | 10.2 | 10.3 |
| Du et al., 2025^31^ | Y | Y | Y | Y | Y | Y | Y | Y | N | Y | Y | Y | Y | Y | N | Y | Y | N | Y | Y | Y | Y | Y | Y | Y | Y | Y | Y | Y | Y |
| He et al., 2024^12^ | Y | Y | Y | Y | Y | Y | Y | Y | Y | Y | Y | Y | Y | Y | Y | Y | Y | Y | Y | Y | Y | Y | Y | Y | Y | Y | Y | Y | Y | Y |
| Liu et al., 2024^14^ | Y | Y | Y | Y | Y | Y | Y | Y | N | Y | Y | Y | Y | Y | Y | Y | Y | Y | Y | Y | N | Y | Y | Y | Y | Y | N | Y | Y | Y |
| Liu et al., 2024^15^ | Y | Y | Y | Y | Y | Y | Y | Y | Y | Y | Y | Y | Y | Y | N | Y | Y | Y | Y | Y | Y | Y | Y | Y | Y | Y | Y | Y | Y | Y |
| Liu et al., 2025^29^ | Y | Y | Y | Y | Y | Y | Y | Y | Y | Y | Y | Y | Y | Y | Y | Y | N | Y | N | Y | N | Y | Y | Y | Y | Y | Y | Y | Y | Y |
| Liu et al., 2025^32^ | Y | Y | Y | Y | Y | Y | Y | Y | Y | Y | Y | Y | Y | Y | N | Y | Y | Y | Y | Y | Y | Y | Y | Y | Y | Y | Y | Y | Y | Y |
| Mu et al., 2024^13^ | Y | Y | Y | Y | Y | Y | Y | Y | N | Y | Y | Y | Y | Y | Y | Y | Y | N | Y | Y | Y | Y | Y | Y | Y | Y | Y | Y | Y | Y |
| Shui et al., 2023^30^ | Y | Y | Y | Y | Y | Y | Y | Y | N | Y | Y | Y | Y | Y | N | Y | Y | N | N | Y | N | Y | Y | Y | Y | Y | N | Y | Y | Y |
| Wang, 2025^1^ | Y | Y | Y | Y | Y | Y | Y | Y | N | Y | Y | Y | Y | Y | Y | Y | Y | Y | Y | Y | Y | Y | Y | Y | Y | Y | Y | Y | Y | Y |
| Yang et al., 2025^11^ | Y | Y | Y | Y | Y | Y | Y | Y | N | Y | Y | Y | Y | Y | Y | Y | Y | Y | Y | Y | Y | Y | Y | Y | Y | Y | Y | Y | Y | Y |
| Zhang, 2025^33^ | Y | Y | Y | Y | Y | Y | Y | Y | N | Y | Y | Y | Y | Y | N | Y | Y | Y | Y | Y | Y | Y | Y | Y | Y | Y | Y | Y | Y | Y |
| Note: Y: Yes; N: No. | | | | | | | | | | | | | | | | | | | | | | | | | | | | | | |

| Supplemental Table 2. The classification of attributes included in the study and their relative importance and ranking | | | | | |
| --- | --- | --- | --- | --- | --- |
| Author (year) | Attribute | Broad category | Subcategory | Relative  Importance (%) | Rank |
| Du et al. (2025)^31^ | Payment costs | Economic guarantee | Payment costs | - | 1 |
|  | Evaluation of platform services | Service quality guarantee | User ratings | - | 2 |
|  | Service comprehensiveness | Service quality guarantee | Service content | - | 3 |
|  | Nurse qualification | Professional competence guarantee | Professional qualifications | - | 4 |
|  | Service approach | Work condition guarantee | Working mode | - | 5 |
|  | Service wait time | Service efficiency guarantee | Service accessibility | - | 6 |
| He et al. (2024)^12^ | Income per visit | Economic guarantee | Service revenue | 41.93 | 1 |
|  | Personal safety management | Service quality guarantee | Service safety | 27.00 | 2 |
|  | Duration per visit | Service efficiency guarantee | Service duration | 16.05 | 3 |
|  | Medical risk prevention | Service quality guarantee | Service safety | 10.16 | 4 |
|  | Refresher training | Work condition guarantee | Continuing education | 2.53 | 5 |
|  | Working modes | Work condition guarantee | Working mode | 2.33 | 6 |
| Liu et al. (2024)^14^ | Character of structure | Service foundation guarantee | Service provider | - | 1 |
|  | Payment costs | Economic guarantee | Payment costs | - | 2 |
|  | Reservation method | Service efficiency guarantee | Reservation method | - | 3 |
|  | Service content | Service quality guarantee | Service content | - | 4 |
|  | Service decision-making | Humanistic care guarantee | Subjects of decision | - | 5 |
|  | Nurses’ experience | Professional competence guarantee | Professional qualifications | - | 6 |
| Liu et al. (2024)^15^ | Medical insurance reimbursement rate | Economic guarantee | Reimbursement ratio | 26.44 | 1 |
|  | Hospital size | Service foundation guarantee | Organization size | 17.24 | 2 |
|  | Service provider | Professional competence guarantee | Professional qualifications | 17.04 | 3 |
|  | Service platform | Service foundation guarantee | Service provider | 16.21 | 4 |
|  | Single service cost | Economic guarantee | Payment costs | 13.29 | 5 |
|  | Services content | Humanistic care guarantee | Individualized service | 9.78 | 6 |
| Liu et al. (2025)^29^ | Service costs | Economic guarantee | Service revenue | 37.74 | 1 |
|  | Incentive mechanism | Work condition guarantee | Organizational incentives | 24.33 | 2 |
|  | Service mode | Work condition guarantee | Working mode | 17.90 | 3 |
|  | Service platform | Service foundation guarantee | Service provider | 13.56 | 4 |
|  | Service content | Humanistic care guarantee | Individualized service | 6.47 | 5 |
| Liu et al. (2025)^32^ | Service costs | Economic guarantee | Payment costs | 19.7 | 1 |
|  | Hospital size | Service foundation guarantee | Organization size | 19.6 | 2 |
|  | Service provider | Professional competence guarantee | Professional qualifications | 18.7 | 3 |
|  | Reimbursement ratio | Economic guarantee | Reimbursement ratio | 17.0 | 4 |
|  | Service platform | Service foundation guarantee | Service provider | 12.9 | 5 |
|  | Service content | Humanistic care guarantee | Individualized service | 11.7 | 6 |
| Mu et al. (2024)^13^ | Service costs | Economic guarantee | Payment costs | 23 | 1 |
|  | Nurse qualification | Professional competence guarantee | Professional qualifications | 21 | 2 |
|  | Evaluation of platform services | Service quality guarantee | User ratings | 21 | 2 |
|  | Response time | Service efficiency guarantee | Service accessibility | 16 | 3 |
|  | Service safety | Service quality guarantee | Service safety | 8 | 4 |
|  | Strength of hospital supervision | Service quality guarantee | Service supervision | 7 | 5 |
|  | Health guidance and follow-up | Humanistic care guarantee | Continuing care | 5 | 6 |
| Shui et al. (2023)^30^ | Service distance | Service efficiency guarantee | Service accessibility | - | 1 |
|  | Service revenue | Economic guarantee | Service revenue | - | 2 |
|  | Living status | Humanistic care guarantee | Living arrangement | - | 3 |
|  | Service content | Service quality guarantee | Service content | - | 4 |
|  | Service continuity | Humanistic care guarantee | Continuing care | - | 5 |
| Wang (2025)^1^ | Task complexity | Work condition guarantee | Task complexity | 28.07 | 1 |
|  | Business consideration | Economic guarantee | Service revenue | 26.22 | 2 |
|  | Service safety | Service quality guarantee | Service safety | 21.61 | 3 |
|  | Service distance | Service efficiency guarantee | Service accessibility | 15.94 | 4 |
|  | Nursing operation hours | Service efficiency guarantee | Service duration | 7.25 | 5 |
|  | Service time | Service efficiency guarantee | Service time slots | Not statistically significant | |
| Yang et al. (2025)^11^ | Safety and security | Service quality guarantee | Service safety | 30.89 | 1 |
|  | Income | Economic guarantee | Service revenue | 27.41 | 2 |
|  | Patient and family cooperation | Work condition guarantee | Patient and family cooperation | 18.47 | 3 |
|  | Service type | Service quality guarantee | Service content | 11.96 | 4 |
|  | Commute time | Service efficiency guarantee | Service accessibility | 11.27 | 5 |
| Zhang (2025)^33^ | Quality of life | Humanistic care guarantee | Quality of life | - | 1 |
|  | Source of payment | Economic guarantee | Payment method | - | 2 |
|  | Waiting period for post-booking care | Service efficiency guarantee | Service accessibility | - | 3 |
|  | Service content | Humanistic care guarantee | Individualized service | - | 4 |
|  | Service costs | Economic guarantee | Payment costs | - | 5 |

| Supplemental Table 3. Frequencies of attribute importance ranks by attribute type (full list) | | | | | |
| --- | --- | --- | --- | --- | --- |
| Attribute sub-categories | Frequencies of attribute importance ranks | | | | |
|  | Rank 1 | Rank 2 | Rank 3 | Rank 4 | Rank 5+ |
| Payment costs | 3 | 1 | 0 | 0 | 2 |
| Service revenue | 2 | 3 | 0 | 0 | 0 |
| Service safety | 1 | 1 | 1 | 2 | 0 |
| Service accessibility | 1 | 0 | 2 | 1 | 2 |
| Service provider | 1 | 0 | 0 | 2 | 1 |
| Reimbursement ratio | 1 | 0 | 0 | 1 | 0 |
| Task complexity | 1 | 0 | 0 | 0 | 0 |
| Quality of life | 1 | 0 | 0 | 0 | 0 |
| User ratings | 0 | 2 | 0 | 0 | 0 |
| Organization size | 0 | 2 | 0 | 0 | 0 |
| Professional qualifications | 0 | 1 | 2 | 1 | 1 |
| Organizational incentives | 0 | 1 | 0 | 0 | 0 |
| Payment method | 0 | 1 | 0 | 0 | 0 |
| Service content | 0 | 0 | 1 | 3 | 0 |
| Service duration | 0 | 0 | 1 | 0 | 1 |
| Working mode | 0 | 0 | 1 | 0 | 2 |
| Reservation method | 0 | 0 | 1 | 0 | 0 |
| Living arrangement | 0 | 0 | 1 | 0 | 0 |
| Patient and family cooperation | 0 | 0 | 1 | 0 | 0 |
| Individualized service | 0 | 0 | 0 | 1 | 3 |
| Continuing care | 0 | 0 | 0 | 0 | 2 |
| Continuing education | 0 | 0 | 0 | 0 | 1 |
| Subjects of decision | 0 | 0 | 0 | 0 | 1 |
| Service supervision | 0 | 0 | 0 | 0 | 1 |
| Service time slots | - | - | - | - | - |
| Note: Service time slots were not assigned an importance rank because the corresponding attribute was not statistically significant. | | | | | |

| Supplemental Table 4. Frequencies of attribute importance ranks by attribute type (patient’s perspective) | | | | | |
| --- | --- | --- | --- | --- | --- |
| Attribute sub-categories | Frequencies of attribute importance ranks | | | | |
|  | Rank 1 | Rank 2 | Rank 3 | Rank 4 | Rank 5+ |
| Payment costs | 3 | 1 |  |  | 2 |
| Service provider | 1 |  |  | 1 | 1 |
| Reimbursement ratio | 1 |  |  | 1 |  |
| Quality of life | 1 |  |  |  |  |
| User ratings |  | 2 |  |  |  |
| Organization size |  | 2 |  |  |  |
| Professional qualifications |  | 1 | 2 | 1 | 1 |
| Payment method |  | 1 |  |  |  |
| Service accessibility |  |  | 2 |  | 1 |
| Reservation method |  |  | 1 |  |  |
| Service content |  |  | 1 | 1 |  |
| Individualized service |  |  |  | 1 | 2 |
| Service safety |  |  |  | 1 |  |
| Subjects of decision |  |  |  |  | 1 |
| Working mode |  |  |  |  | 1 |
| Service supervision |  |  |  |  | 1 |
| Continuing care |  |  |  |  | 1 |

| Supplemental Table 5. Frequencies of attribute importance ranks by attribute type (nurse’s perspective) | | | | | |
| --- | --- | --- | --- | --- | --- |
| Attribute sub-categories | Frequencies of attribute importance ranks | | | | |
|  | Rank 1 | Rank 2 | Rank 3 | Rank 4 | Rank 5+ |
| Service revenue | 2 | 3 |  |  |  |
| Service safety | 1 | 1 | 1 | 1 |  |
| Service accessibility | 1 |  |  | 1 | 1 |
| Task complexity | 1 |  |  |  |  |
| Organizational incentives |  | 1 |  |  |  |
| Service duration |  |  | 1 |  | 1 |
| Working mode |  |  | 1 |  | 1 |
| Living arrangement |  |  | 1 |  |  |
| Patient and family cooperation |  |  | 1 |  |  |
| Service content |  |  |  | 2 |  |
| Service provider |  |  |  | 1 |  |
| Continuing education |  |  |  |  | 1 |
| Individualized service |  |  |  |  | 1 |
| Continuing care |  |  |  |  | 1 |

| Supplemental Table 6. Summary of preference heterogeneity reported in the included studies | | | | |
| --- | --- | --- | --- | --- |
| Author (year) | Variable | Main finding | Effect estimate | Statistical significance |
| Yang et al. (2025)^11^ | Professional title | Senior nurses placed greater emphasis on service safety. | β = 1.421 vs. 0.725 | P < 0.05 |
|  | Marital status | Unmarried nurses placed greater emphasis on patient/family cooperation. | β = 1.105 vs. 0.314 | P < 0.05 |
| Wang (2025)^1^ | Professional title | Higher-title nurses placed greater emphasis on service safety. | β = 0.807 vs. 0.225 | P < 0.05 |
|  | Professional title | Junior nurses placed greater emphasis on nursing task complexity. | β = 0.898 vs. 0.669 | P < 0.001 |
|  | Years of experience | Nurses with longer working experience placed greater emphasis on service safety. | β = 0.831 vs. 0.157 | P < 0.001 |
|  | Years of experience | Nurses with shorter working experience placed greater emphasis on nursing task complexity and service distance. | Task complexity: β = 0.871 vs. 0.656 Service distance: β = 0.449 vs. 0.384 | P < 0.001 |
|  | Income | Higher-income nurses placed greater emphasis on service safety. | β = 1.221 vs. 0.147 | P < 0.001 |
|  | Income | Lower-income nurses placed greater emphasis on nursing task complexity. | β = 0.820 vs. 0.682 | P < 0.001 |
| Liu et al. (2024)^14^ | Educational attainment | Higher-educated participants showed a stronger preference for services provided by public institutions. | Interaction β = −0.988 (non-public vs. public institutions) | P < 0.05 |
|  | Income | Higher-income participants showed a stronger preference for services provided by public institutions. | Interaction β = −1.368 (non-public vs. public institutions) | P < 0.05 |
| Du et al. (2025)^31^ | Educational attainment | Participants with lower educational attainment showed larger preference coefficients for comprehensive service content. | β = 0.934 vs. 0.519 | P < 0.05 |
|  | Educational attainment | Participants with lower educational attainment showed larger preference coefficients for platform service ratings. | β = 1.655 vs. 0.887 | P < 0.05 |

| Supplemental Table 7. Marginal willingness-to-pay estimates | | | | |
| --- | --- | --- | --- | --- |
| Author (year) | Data Collection Year | Attribute | Level | WTP estimate (95% CI)  (2025 Price Level, CNY) |
| Du et al. (2025)^31^ | 2024 | Nurse qualification | General nurse | Reference |
|  |  |  | Chinese medicine nurse specialist | 24.22 (11.11, 37.32) |
|  |  | Service approach | Remote service | Reference |
|  |  |  | Door-to-door service | 7.40 (-7.41, 22.23) |
|  |  |  | Remote service + door-to-door service | 10.80 (-2.73, 24.33) |
|  |  | Service wait time | 60min | Reference |
|  |  |  | 30min | 8.36 (-3.57, 20.29) |
|  |  | Service comprehensiveness | General | Reference |
|  |  |  | Comprehensive | 18.03 (4.38, 31.68) |
|  |  |  | Very comprehensive | 26.75 (10.55, 42.95) |
|  |  | Evaluation of platform services | Ordinary | Reference |
|  |  |  | Good | 40.17 (24.89, 55.46) |
|  |  |  | Excellent | 55.68 (36.70, 74.66) |
| He et al. (2024)^12^ | 2022 | Working mode | Full-time → part-time | -4.00 (NR) |
|  |  | Duration per visit | 4h → 1h | -9.19 (NR) |
|  |  | Personal safety management | Basic → superior | -46.36 (NR) |
|  |  | Medical risk prevention | Basic → superior | -17.44 (NR) |
|  |  | Refresher training | More than 8h → less than 4 h | 4.35 (NR) |
| Liu et al. (2024)^14^ | 2023 | Reservation method | Offline reservation → both online and offline | 40.61 (NR) |
|  |  | Service content | Basic care package → health management packages | -30.65 (NR) |
|  |  | Character of structure | Public institutions → non-public institutions | -173.36 (NR) |
| Liu et al. (2024)^15^ | 2023 | Medical insurance reimbursement rate | 30% → 70% | 203.31 (41.68, 364.93) |
|  |  | Hospital size | Small → large | 155.91 (55.31, 256.61) |
|  |  | Service provider | Primary nurse → intermediate nurse | 128.46 (34.17, 222.64) |
|  |  | Service platform | Enterprise-led → hospital-led | 127.86 (16.63, 239.08) |
|  |  |  | Enterprise-led → government-led | 121.84 (18.74, 225.05) |
|  |  | Services content | Basic package → personality package service | -136.27 (-251.60, -20.84) |
| Liu et al. (2025)^29^ | 2023 | Incentive mechanism | None → integration into performance appraisal | -130.90 （-161.27, -100.54） |
|  |  |  | None → priority in title promotion | -120.48 (-148.95, -92.01) |
|  |  |  | None → priority given to further education and training | -52.74 (-80.34, -25.13) |
|  |  | Service mode | Individual home visits → team home service | -107.81 (-127.90, -87.72) |
|  |  | Service platform | Business-led → government-led | -73.91 (-91.68, -56.13) |
|  |  | Service content | Basic package → personalized package | -33.59 (-50.19, -16.98) |
| Liu et al. (2025)^32^ | 2023 | Service provider | Primary nurse → advanced nurse | 165.43 (87.57, 243.29) |
|  |  |  | Primary nurse → intermediate nurses | 67.23 (21.64, 112.83) |
|  |  | Service content | Basic package → personalized package | -86.97 (-135.07, -38.98) |
|  |  | Reimbursement ratio | 30% → 50% | 96.69 (23.25, 170.04) |
|  |  |  | 30% → 70% | 135.47 (57.31, 213.53) |
|  |  | Service platform | Business-led → hospital-led | 93.99 (41.38, 146.69) |
|  |  |  | Business-led → government-led | 101.90 (45.69, 158.12) |
|  |  | Hospital size | Small → medium | 118.94 (53.81, 184.17) |
|  |  |  | Small → large | 161.32 (97.69, 224.95) |
| Mu et al. (2024)^13^ | 2023 | Nurse qualification | 2 years-5 years | Reference |
|  |  |  | 6 years ~ 10 | 90.78 (51.89, 129.71) |
|  |  |  | ＞10 | 152.00 (101.19, 202.78) |
|  |  | Response time | 90 min | Reference |
|  |  |  | 30 min | 152.00 (105.22, 198.80) |
|  |  |  | 60 min | 86.57 (49.54, 123.56) |
|  |  | Evaluation of platform services | Average | Reference |
|  |  |  | Good | 116.43 (77.44, 155.38) |
|  |  |  | Very good | 288.78 (221.17, 356.43) |
|  |  | Health guidance and follow-up | Not available | Reference |
|  |  |  | Provide | 74.95 (38.54, 111.36) |
|  |  | Service safety | Buying accident insurance | Reference |
|  |  |  | Equipped with care recorders | 36.67 (16.13, 75.55) |
|  |  |  | Double-person door-to-door service | 62.73 (26.15, 99.35) |
|  |  | Strength of hospital supervision | Small | Reference |
|  |  |  | Large | 125.45 (78.18, 172.69) |
| Shui et al. (2023)^30^ | 2022 | NR | | |
| Wang (2025)^1^ | 2024 | Service distance | ＞ 6 km | Reference |
|  |  |  | ＜ 3 km | -24.31 (-31.95, -16.67) |
|  |  |  | ＞3 km-6 km | -11.34 (-17.47, -5.21) |
|  |  | Service time | Nurses’ rest day mornings | Reference |
|  |  |  | Nurses’ rest day afternoons | -1.38 (-6.96, 4.19) |
|  |  | Task complexity | Difficult | Reference |
|  |  |  | Easy | -42.82 (-52.89, -32.75) |
|  |  |  | Medium | -34.23 (-42.46, -26.00) |
|  |  | Nursing operation hours | ＞ 30 min | Reference |
|  |  |  | ＜ 15 min | -11.05 (-17.49, -4.61) |
|  |  |  | 15 min-30 min | -7.61 (-13.57, -1.65) |
|  |  | Service safety | Normal | Reference |
|  |  |  | Good | -32.97 (-43.04, -22.89) |
|  |  |  | Medium | -14.15 (-20.73, -7.57) |
| Yang et al. (2025)^11^ | 2021 | Patient and family cooperation | Poor cooperation → moderate cooperation | 1123.86 (622.77, 1669.06) |
|  |  |  | Poor cooperation → good cooperation | 1661.45 (1170.29, 2313.09) |
|  |  | Safety and security | Low security → medium security | 1822.33 (1345.24, 2592.01) |
|  |  |  | Low security → high security | 2648.42 (1879.54, 3680.93) |
|  |  | Commute time | ＞ 30 min → ≤ 30 min | 1058.28 (663.37, 1558.97) |
|  |  | Service type | Invasive operations → non-invasive operations | 865.55 (271.19, 1558.77) |
| Zhang (2025)^33^ | 2024 | Source of payment | Self-paying | Reference |
|  |  |  | Medical insurance | 114.94 (76.07, 153.80) |
|  |  |  | Commercial insurance | 111.91 (67.63, 156.20) |
|  |  |  | Other sources | 96.94 (58.48, 135.39) |
|  |  | Service content | Basic services + hospice preparation + grief counseling | Reference |
|  |  |  | Basic services + life (death) education + advance living wills | 17.38 (-14.80, 49.55) |
|  |  |  | Basic services + grief counseling + family care support | 32.18 (-0.83, 65.19) |
|  |  | Quality of life | Bad | Reference |
|  |  |  | General | 164.14 (130.66, 197.62) |
|  |  |  | Good | 252.45 (197.91, 306.98) |
|  |  | Waiting period for post-booking care | Waiting time 25 h-48 h | Reference |
|  |  |  | Waiting time 6 h-12 h | 106.46 (69.90, 143.02) |
|  |  |  | Waiting time 13 h-24 h | 68.86 (35.86, 101.85) |
| Note: WTP estimates and 95% confidence intervals were converted to the 2025 price level using the Consumer Price Index (CPI) reported by the National Bureau of Statistics of China. Conversion factors were 2024=1.000, 2023=1.002, 2022=1.004, and 2021=1.024. Negative WTP indicates a negative preference for attribute changes, implying that patients require compensation or nurses accept reduced income for preferred changes. CNY: Chinese yuan. NR: not reported. | | | | |

Supplemental Figure 1 (A). Frequencies of attribute importance ranks by respondent perspective (patient)

Supplemental Figure 1 (B). Frequencies of attribute importance ranks by respondent perspective (nurse)
