# Supplementary material for: Preferences for Internet Plus Nursing Services Among Nurses and Patients in China: A Systematic Review of Discrete Choice Experiments
Source: J Nurs Manag. 2026 Oct 4;2026:8852180. doi: 10.1155/jonm/8852180 (PMC13635678; doi:10.1155/jonm/8852180)
Supplement: Supplementary file 1 [file JONM-2026-8852180-s001.zip › Supplemental Appendix B Search Strategy and Search History.docx]

**Supplemental Appendix B: Search Strategy and Search History**

PubMed:

| **#** | **Searches** | **Results** |
| --- | --- | --- |
| #1 | ("Patient Preference"[MeSH Terms]) OR ("discrete choice experiment*"[All Fields] OR "DCE"[All Fields]) OR ("choice experiment*"[All Fields] OR "choice model*"[All Fields] OR "choice modelling"[All Fields] OR "choice modeling"[All Fields]) OR ("discrete choice analysis"[All Fields] OR "discrete choice model*"[All Fields]) OR ("conjoint analys*"[All Fields] OR "conjoint choice experiment*"[All Fields] OR "choice-based conjoint"[All Fields] OR "choice based conjoint"[All Fields]) OR ("stated preference*"[All Fields] OR "stated choice*"[All Fields] OR "stated-preference method*"[All Fields] OR "preference elicitation*"[All Fields]) OR ("best-worst scaling"[All Fields] OR "BWS"[All Fields] OR "maximum difference scaling"[All Fields] OR "maxdiff"[All Fields]) | 32,613 |
| #2 | (("Internet plus nursing service*" [All Fields]) OR ("Internet"[MeSH] OR "Internet"[All Fields]) OR ("Telemedicine"[MeSH] OR "Telemedicine"[All Fields] OR "Telehealth"[All Fields] OR "Telenursing"[All Fields]) OR ("Online"[All Fields] OR "Virtual"[All Fields] OR "Remote"[All Fields] OR "Web-Based"[All Fields]) OR ("Digital health"[All Fields] OR "Mobile health"[All Fields] OR "MHealth"[All Fields] OR "eHealth"[All Fields]) OR ("Platform*"[All Fields])) AND (("Home Care Services"[MeSH] OR "Home Nursing"[MeSH]) OR ("Nursing"[All Fields] OR "Nurses"[All Fields]) OR ("door-to-door service*"[All Fields] OR "door to door service*"[All Fields]) OR ("Community Health Nursing"[MeSH] OR "Community Nursing"[All Fields]) OR ("home care"[All Fields] OR "domiciliary care"[All Fields] OR "visiting nurse"[All Fields])) | 82,623 |
| #3 | #1 AND #2 | 352 |

Embase:

| **#** | **Searches** | **Results** |
| --- | --- | --- |
| #1 | 'patient preference'/exp OR 'patient preference' OR 'discrete choice experiment*':ab,ti OR 'dce':ab,ti OR 'choice experiment*':ab,ti OR 'choice model*':ab,ti OR 'choice modelling':ab,ti OR 'choice modeling':ab,ti OR 'discrete choice analysis':ab,ti OR 'discrete choice model*':ab,ti OR 'conjoint analys*':ab,ti OR 'conjoint choice experiment*':ab,ti OR 'choice-based conjoint':ab,ti OR 'choice based conjoint':ab,ti OR 'stated preference*':ab,ti OR 'stated choice*':ab,ti OR 'stated-preference method*':ab,ti OR 'preference elicitation*':ab,ti OR 'best-worst scaling':ab,ti OR 'bws':ab,ti OR 'maximum difference scaling':ab,ti OR 'maxdiff':ab,ti | 61,276 |
| #2 | ('internet plus nursing service*':ab,ti OR 'internet'/exp OR internet:ab,ti OR 'telemedicine'/exp OR 'telemedicine':ab,ti OR 'telehealth':ab,ti OR 'telenursing':ab,ti OR 'online':ab,ti OR 'virtual':ab,ti OR 'remote':ab,ti OR 'web-based':ab,ti OR 'digital health':ab,ti OR 'mobile health':ab,ti OR 'mhealth':ab,ti OR 'ehealth':ab,ti OR 'platform*':ab,ti) AND ('home care service'/exp OR 'home nursing'/exp OR 'community health nursing'/exp OR 'postdischarge care' OR 'discharge planning'/exp OR 'nursing':ab,ti OR 'nurses':ab,ti OR 'door-to-door service*':ab,ti OR 'door to door service*':ab,ti OR 'community nursing':ab,ti OR 'home care':ab,ti OR 'domiciliary care':ab,ti OR 'visiting nurse':ab,ti) | 65,182 |
| #3 | #1 AND #2 | 507 |

Scopus:

| **#** | **Searches** | **Results** |
| --- | --- | --- |
| #1 | TITLE-ABS-KEY ( "discrete choice experiment*" OR DCE OR "choice experiment*" OR "choice model*" OR "choice modelling" OR "choice modeling" OR "discrete choice analysis" OR "discrete choice model*" OR "conjoint analys*" OR "conjoint choice experiment*" OR "choice-based conjoint" OR "choice based conjoint" OR "stated preference*" OR "stated choice*" OR "stated-preference method*" OR "preference elicitation*" OR "best-worst scaling" OR BWS OR "maximum difference scaling" OR maxdiff ) | 61,810 |
| #2 | ( TITLE-ABS-KEY ( "Internet plus nursing service*" OR "Internet plus nursing service program*" OR "Internet plus nursing services*" OR "Internet nursing service*" OR telehealth OR telenursing OR eHealth OR mHealth OR "digital health" ) AND TITLE-ABS-KEY ( "home nursing" OR "home care" OR nursing OR nurse* OR "community nursing" OR "visiting nurse" ) ) | 10,175 |
| #3 | #1 AND #2 | 9 |

CINAHL Complete:

| **#** | **Searches** | **Results** |
| --- | --- | --- |
| #1 | (MH "Patient Preference") OR SU (discrete choice experiment* OR DCE OR choice experiment* OR choice model* OR choice modelling OR choice modeling OR discrete choice analysis OR discrete choice model* OR conjoint analys* OR conjoint choice experiment* OR choice-based conjoint OR choice based conjoint OR stated preference* OR stated choice* OR stated-preference method* OR preference elicitation* OR best-worst scaling OR BWS OR maximum difference scaling OR maxdiff) | 5,843 |
| #2 | ((MH "Internet") OR (MH "Telemedicine") OR SU ("Internet plus nursing service*" OR "Internet" OR "Telemedicine" OR "Telehealth" OR "Telenursing" OR "Online" OR "Virtual" OR "Remote" OR "Web-Based" OR "Digital health" OR "Mobile health" OR "MHealth" OR "eHealth" OR "Platform*")) AND ((MH "Home Care Services") OR (MH "Home Nursing") OR (MH "Community Health Nursing") OR (MH "Postdischarge Care") OR (MH "Discharge Planning") OR SU ("Nursing" OR "Nurses" OR "door-to-door service*" OR "door to door service*" OR "Community Nursing" OR "home care" OR "domiciliary care" OR "visiting nurse")) | 15,588 |
| #3 | #1 AND #2 | 27 |

PsycInfo:

| **#** | **Searches** | **Results** |
| --- | --- | --- |
| #1 | (Patient Preference or discrete choice experiment* or DCE or choice experiment* or choice model* or choice modelling or choice modeling or discrete choice analysis or discrete choice model* or conjoint analys* or conjoint choice experiment* or choice-based conjoint or choice based conjoint or stated preference* or stated choice* or stated-preference method* or preference elicitation* or best-worst scaling or BWS or maximum difference scaling or maxdiff).ab. | 6,778 |
| #2 | ((Internet plus nursing service* or Internet or Telemedicine or Telehealth or Telenursing or Online or Virtual or Remote or Web-Based or Digital health or Mobile health or MHealth or eHealth or Platform*) and (Home Care Services or Home Nursing or Nursing or Nurses or door-to-door service* or door to door service* or Community Health Nursing or Community Nursing or Postdischarge Care or Discharge Planning or home care or domiciliary care or visiting nurse)).ab. | 8,153 |
| #3 | #1 AND #2 | 13 |

China National Knowledge Infrastructure:

| **#** | **Searches** | **Results** |
| --- | --- | --- |
| #1 | （主题： 离散选择试验 + 离散选择实验 + DCE + 偏好）AND（主题： 互联网＋护理 + 互联网护理 + 网约护理 + 上门护理 + 家庭护理） | 18 |

WanFang Data:

| **#** | **Searches** | **Results** |
| --- | --- | --- |
| #1 | 主题:(离散选择试验 OR 离散选择实验 OR DCE) and 主题:(互联网护理 OR 互联网 OR 网约护理 OR 上门护理 OR 家庭护理) | 452 |

Weipu Database:

| **#** | **Searches** | **Results** |
| --- | --- | --- |
| #1 | 任意字段=离散选择试验 OR 离散选择实验 OR DCE AND 任意字段=互联网护理 OR 互联网 OR 网约护理 OR 上门护理 OR 家庭护理 | 30 |
